# Thromboembolic disease in COVID-19 patients: A brief narrative review

**DOI:** 10.1186/s40560-020-00483-y

**Published:** 2020-09-14

**Authors:** Samhati Mondal, Ashley L. Quintili, Kunal Karamchandani, Somnath Bose

**Affiliations:** 1 Department of Anesthesiology, University of Maryland School of Medicine, Baltimore, MD USA; 2 Department of Pharmacy, Penn State Health Milton S. Hershey Medical Center, Hershey, PA USA; 3 Department of Anesthesiology and Perioperative Medicine, Penn State Health Milton S. Hershey Medical Center, Hershey, PA USA; 4 Department of Anesthesiology, Critical Care and Pain Medicine, Beth Israel Deaconess Medical Center, Harvard Medical School, One Deaconess Road, Rosenberg 470, Boston, MA 02215 USA

**Keywords:** COVID-19, thrombosis, thromboembolism, SARS-CoV2, management, coagulation

## Abstract

Corona virus 2 (SARS-CoV2/ Severe Acute Respiratory Syndrome Corona Virus 2) infection has emerged as a global health crisis. Incidence of thromboembolic disease is reported to be high in SARS-CoV2 disease and is seen in a multitude of organ systems ranging from cutaneous thrombosis to pulmonary embolism, stroke or coronary thrombosis sometimes with catastrophic outcomes. Evidence points towards a key role of thromboembolism, hypercoagulability and over production of proinflammatory cytokines mimicking a “cytokine storm” which leads to multiorgan failure. This brief narrative review highlights the pathophysiology and risk factors of thromboembolic disease and provides a framework for management of anticoagulation based on the current evidence.

## Introduction

The current coronavirus pandemic caused by the SARS-CoV2 has rapidly emerged as a global health crisis. To date, over four million people have been affected by coronavirus disease 2019 (COVID-19) worldwide in about 188 countries and the number continues to grow [1]. In the United States alone, confirmed cases and deaths continue to rise, with current estimates at more than 1.9 million positive patients and over 110,000 deaths [1]. Symptoms range from asymptomatic or mild constitutional symptoms to pneumonia, sepsis and sometimes severe acute respiratory distress syndrome (ARDS) necessitating hospitalization and intensive care unit (ICU) admission [2]. The pivotal role of thrombo-inflammation and endothelial injury in the pathogenesis of the disease is being increasingly recognized. Overproduction of pro-inflammatory cytokines, including tumor necrosis factor (TNF), Interleukin (IL) -6, IL-8, and IL-1β, is believed to be the cause of what is being termed, “cytokine release syndrome” or “cytokine storm”, a phenomenon which is however not unique to this disease and has been noted in sepsis and sterile inflammation as well. This exaggerated cytokine response may lead to multiorgan failure and eventually death in some patients [3]. In addition to elevations in pro-inflammatory markers, hypercoagulability has been identified to be playing a key role determining prognosis in patients with COVID-19 [4]. In some observational series, thrombotic complications have been noted to be as high as 31% in patients requiring ICU admission and the risk persists even in patients on anticoagulation [5–8].

We searched for all published, readily accessible, peer-reviewed, full articles written in English on PUBMED and EMBASE (between December 1^st^, 2019 to June 6^th^, 2020) related to thromboembolic complications seen in COVID -19 before writing this review. Majority of the articles included retrospective, observational, single or multicenter studies or case reports and correspondences.

In this brief narrative review, we discuss the pathophysiological mechanisms, clinical manifestations of thrombotic complications noted in patients with COVID-19 and describe a pragmatic approach for management of anticoagulation strategies in these patients based on the currently available evidence.

### Pathogenesis and risk factors

COVID-19 shares multiple similarities with other well defined inflammatory states such as sepsis and sterile inflammation wherein simultaneous rise in pro and anti-inflammatory cytokines are seen [9, 10]. More pertinently, there is evidence of complement activation in COVID-19 by direct endothelial infection which includes release of anaphylotoxin C5a [11]. Complement activation as seen in COVID-19 not only drives neutrophil dysfunction leading to susceptibility to secondary infections but also activates the coagulation system thereby propagating a prothrombotic state. Coagulopathy associated with COVID-19 may be explained by the ‘two way activation’ theory, as seen by thrombocytopenia in critically ill patients (TICP) and the encompassing inflammatory and micro-thrombogenic responses that occur when endothelial insult takes place [12]. While the inflammatory pathway releases cytokines, the activation of microthrombotic pathway is mediated by release of large polymers of Von Willebrand factors (VWF). In the face of sepsis- induced endothelial injury, this reaction is aggravated causing enhanced platelet activation and consumption thrombocytopenia [13]. In contrast to the typical consumptive coagulopathy and disseminated intravascular coagulation (DIC) profile observed in sepsis, patients with COVID-19 typically have relatively normal coagulation and platelet profiles. Progression to DIC occurs in a minority of patients, rarely developing in survivors [4]. Therefore it seems that in keeping with Virchow’s triad, thrombosis is driven both by the activation of coagulation factors and endothelium. In-situ immune-thrombosis plays a key role to be the unifying mechanism explaining the micro and macrothrombotic manifestations of the disease. It should however be emphasized that in-situ microthrombosis has also been demonstrated in pulmonary and systemic tissue beds in ARDS and sepsis and therefore may not necessarily be unique to this population.

In addition to the factors mentioned above, these patients have additional risk factors for increased thrombosis, most notable among those being hypoxia, and immobility (made worse by frequent use of prone positioning) [14–16]. Although not systematically assessed, reduced staffing coupled with isolation precautions which limit frequent position changes and mobility may further predispose patients to a prothrombotic state.

### Clinical manifestations of thrombosis

Since the beginning of the COVID-19 pandemic, serious thrombotic complications have been reported in infected patients especially those that are critically ill [7]. Lung autopsies from patients who died of COVID-19 revealed diffuse alveolar edema, thrombosis, formation of hyaline membrane resembling an ARDS like pattern [16–18]. The term MicroCLOTS (microvascular COVID-19 lung vessels obstructive thromboinflammatory syndrome) secondary to microvascular pulmonary thrombosis has been termed to describe the pulmonary manifestations of the disease [19]. In fact, micro-thrombosis, sometimes progressing to macro-thrombosis is not only limited to the lungs, other tissue beds have also been noted to be susceptible. Increasing reports of thrombotic events, including strokes, pulmonary embolism (PE), as well as, cutaneous and alveolar micro-thrombosis have been noted [18]. Various studies reported a wide range of thromboembolic complications including venous (PE, DVT) as well arterial thrombosis. Microthrombosis in lungs noted as high as 80% in autopsy of fatal COVID -19 [20]. Klok et al reported high incidence of VTE (31%) leading to complications like PE (80%), as well as arterial thrombosis (3.7%) [7].

Table 1 & 2 summarize the various thrombotic complications noted in COVID-19 patients as published as of June 6^th^, 2020 obtained by a literature search on PubMed and EMBASE using combinations of the following MeSH terms: COVID-19, SARS-COV2, novel corona virus, thrombosis, thromboembolic complications, pulmonary embolism.

**Table 1:** Thromboembolic complications with COVID 19 disease reported in single/multicenter studies

Author	Country	Venous thromboembolism - (%)	Pulmonary embolism percentage % or N	Arterial thromboembolism percentage (%)	Stroke percentage (%)	Acute coronary syndrome percentage (%)	Mesenteric/bowel Ischemia percentage (%)	Limb Ischemia percentage (%)
Lodigiani et al [21]	Italy	4.4	2.8		2.5	1.1		
Middeldorp et al [22]	Netherlands	20	6.6					
Helms et al [5]	France		16.7				0.7	0.66
Llitjos [6]	Italy	69	23					
Marone [23]	Italy	53						
Wichmann et al [24]	Germany	53	33					
Cui et al [25]	China	25						
Klok et al [7]	Netherlands	27 49	13.5 87	3.7				
Dolhnikoff et al [20]	Brazil		80 (Autopsy finding)					
Ackermann et al [26]	Germany		57 Microthrombi without luminal occlusion					
Demelo-Rodriguez et al [27]	Spain	14.7						
Zhang et al [28]	China	46.1						
Ren at al [29]	China	85.4						
Stoneham et al [30]	UK	7.7						
Voicu et al [31]	France	46						
Desborough et al [32]	UK	15	8					
Al-Samkari et al [33]	USA	6	N=10	2.8				
Fraisse et al [34]	France	31.6		8.4

**Table 2:** Thromboembolic complications with COVID 19 disease reported as case reports/case series/ correspondences

Author	Country	Venous thromboembolism (N)	Pulmonary embolism numbers (N)	Arterial thromboembolism numbers (N)	Stroke numbers (N)	Acute coronary syndrome numbers (N)	Mesenteric/bowel Ischemia numbers (N)	Limb Ischemia numbers (N)
Le Berre et al [35]	France	1	1	1 (Aortic thrombosis)				
Baldacini M et al [36]	France			1 (Arterial thrombosis)				
Giacomelli et al [37]	Italy			1 (Acute aortic graft thrombosis)				
Lacour et al [38]	France					1		
de Barry et al [39]	France	1					1	
Mulvey et al [40]	USA			5 (Placental intravascular thrombosis)				
Griffin et al [41]	USA		3					
Zhou et al [42]	China	1		1 (Atherosclerosis obliterans)				
Zhou et al [43]	China	1			1			
Dominguez-Erquicia et al [44]	Spain				1			
Martinelli et al [45]	Italy		1					
Bozzani et al [46]	Italy	3						
Poggiali et al [47]	Italy	2	1					
Hughes et al [48]	UK	1 (Cerebral venous sinus thrombosis)						
Kashi et al [49]	France			7				
Garaci et al [50]	Italy	1	1					
Lax et al [51]	Austria		11 (Postmortem autopsy finding)					
Mestres et al [52]	Spain			4				
Gomez-Arbelaez et al [53]	Spain	1	1	2	2			1
Davoodi et al [54]	Iran	1						
Viguier et al [55]	France				1			
Rey et al [56]	Spain					1		
Bhayana et al [57]	USA						2	
Hemasian et al [58]	Paris	1						
Fara et al [59]	USA				3			
Azouz et al [60]	France				1		1	
Nauka et al [61]	USA	1						
Seif et al [62]	Egypt					1		
Morales et al [63]	USA	1	1					
Kaur et al [64]	USA							1
Fox et al [65]	USA		10 (Autopsy finding)					
Hinterseer et al [66]	Germany					1		
Vulliamy et al [67]	UK						1	1
Beccara et al [68]	Italy						1	
Faggiano et al [69]	Italy		7					
Szekely et al [70]	Israel	5						
Harari et al [71]	USA					1		
Andrea et al [72]	Italy							1
La Mura et al [73]	Italy	1 (Portal vein thrombosis)						
Horowitz et al [74]	USA	1 (Clot in transit on tricuspid valve)						
Buja et al [75]	USA		1 Autopsy finding					
Qanadli et al [76]	Switzerland		1					
Vitali et al [77]	Italy		1					
Zayet et al [78]	France				2			
Baergen et al [79]	USA			3 (Placental microthrombosis)				
Malentacchi et al [80]	Italy	1			1			

Table 1 illustrates the observational studies and the most common thrombotic complications noted in these studies were venous thrombosis including PEs. There are also reports of arterial thrombosis including aortic graft thrombosis, mesenteric ischemia, coronary and cerebral thrombosis. Table 2 consists of isolated case reports, case series and correspondences. One unique finding of placental thrombosis has been reported as summarized in Table 2 which warrants further research of vertical transmission [40].

### Monitoring and diagnosis of VTE in critically ill patients with COVID-19

Non-survivors of COVID-19 to have significant increases in fibrinogen degradation products (FDP), d-dimer levels, as well as, prolongation of prothrombin time (PT), with 71.4% meeting diagnostic criteria for DIC [4]. It must however be noted that fibrinogen levels may rise initially as an acute phase reactant and such elevations may not necessarily be specific for COVID-19 [81]. Reports of elevated d-dimer levels and fibrinogen are increasingly prevalent in COVID-19 affected patients; leading many institutions to routinely monitor these values. These elevations appear to correlate with increased levels of inflammatory markers and may be indicators for disease severity in addition to thrombotic risk [82, 83].

A high index of clinical suspicion for thrombotic phenomenon and their sequela is warranted for prompt diagnosis. Clinical signs and symptoms of thrombosis such as cutaneous manifestations (“COVID toe”) [84], overt line thrombosis, arterial or venous clots, unexplained increase in oxygen requirement, or organ dysfunction should raise suspicion and prompt further investigation and/or discussion about therapeutic intervention [7] As new information becomes available, it appears increasingly important to routinely monitor platelet count, PT/aPTT, d-dimer, and fibrinogen to assist in anticipating and managing thrombotic complications. It has been reported that d-dimer levels cutoff of 1.5 μg/mL for predicting venous thromboembolic events has a sensitivity and specificity rate of 85% and 88.5% respectively and a negative predictive value of 94.7% [25]. Nonetheless, decisions for initiation of therapeutic anticoagulation should not be based solely on arbitrary d-dimer levels.

Use of viscoelastrometric tests such as Rotational thromboelastometry (ROTEM) could also be used as an important monitoring tool. Short clot formation time (CFT) on INTEM (type of ROTEM to detect Intrinsic pathway abnormality) and EXTEM (type of ROTEM to detect Extrinsic pathway abnormality) and increased maximum clot firmness (MCF) on INTEM, EXTEM, FIBTEM (type of ROTEM to detect fibrinogen abnormality) indicate hypercoagulation and potential for thrombogenesis [85]. Similarly, Thromboelastography (TEG) directed assessment of hypercoagulation status (short R, K and increased K angle and MA) can be predictive of thromboembolism [86].

Dugar and colleagues recently reported a high incidence of spontaneous echo contrast (SEC) in patients as noted on ultrasound examination of venous system while placing central line which could be precursors for venous thromboembolism (VTE). Their findings suggest the potential role of point-of-care ultrasound (POCUS) as a surveillance tool for early detection of patients at higher risk for thrombotic events [87].

### Management of anticoagulation

The optimal approach to management of anticoagulation in these patients remains unclear in absence of well conducted trials. There remains major uncertainty in the optimal management of immune-thrombosis as commonly seen in COVID-19. Current strategies are heavily influenced by observational reports, case series and empirical institutional protocols. In asymptomatic and mildly symptomatic patients who do not require hospital admission, ambulation should continue to be the mainstay of thromboprophylaxis. It is advisable to institute, at minimum, prophylactic anticoagulation in admitted patients without clinical contraindications [7, 88]. Unfractionated heparin and low molecular weight heparin (LMWH) have been successfully used in these patients both prophylactically and therapeutically [89, 90]. Higher doses should be considered for those with higher risk patients (eg, obese, active malignancy, prolonged immobility or recent surgery). As a caveat, it must be noted a high incidence of VTE has been noted even on patients on either prophylactic and therapeutic anticoagulation which makes routine surveillance extremely important [5, 6]**.**

**Fig. 1:**
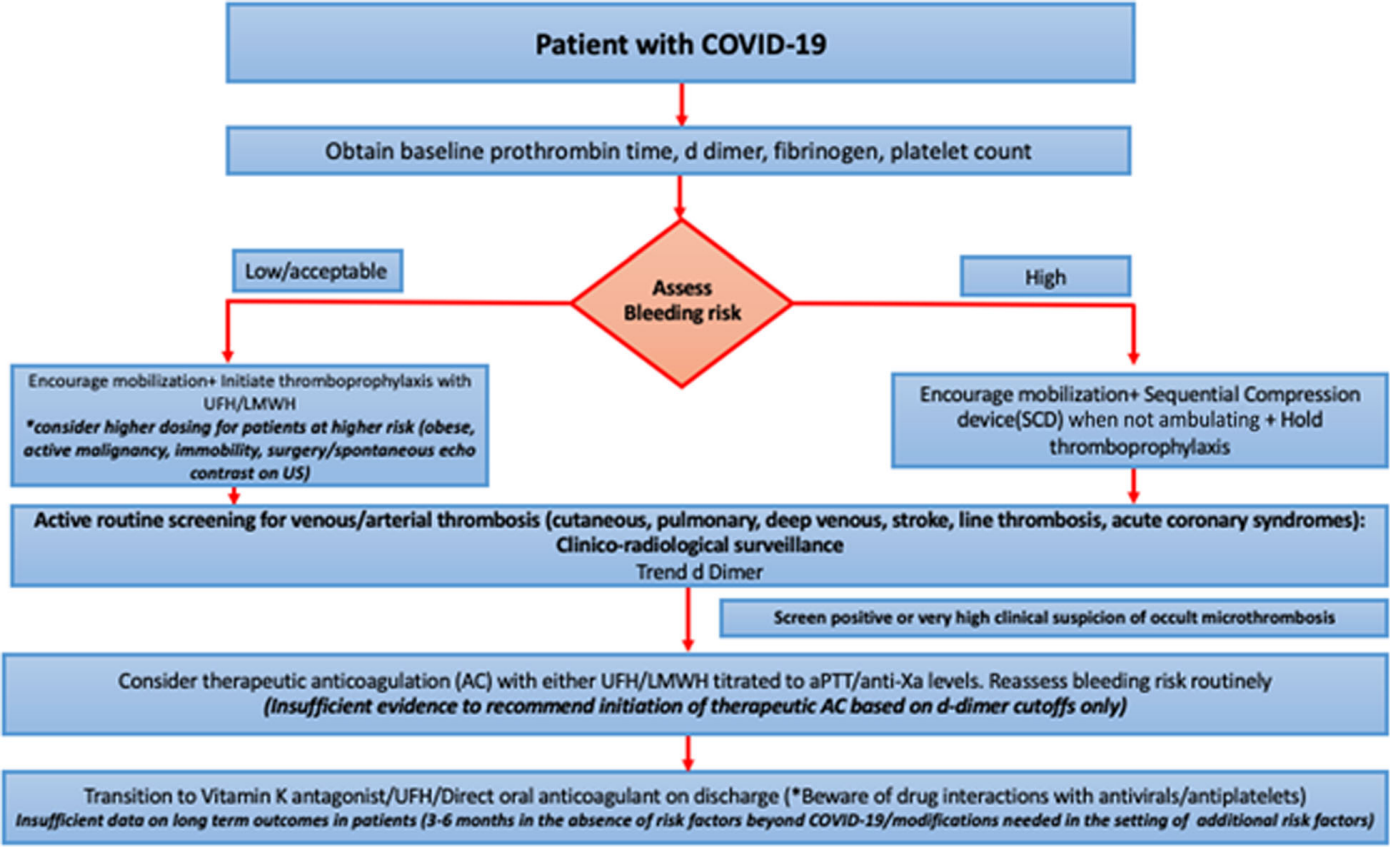
Algorithm for the management of anticoagulation in patients hospitalized with COVID-19

In addition to the usual indications such as obesity or active malignancy where higher intensity dosing of prophylactic anticoagulation may be warranted, patients with COVID-19 who demonstrate SEC on surveillance imaging may be considered for augmented dosing although high quality data to support routine use of this strategy is currently unavailable. Although some retrospective studies have demonstrated systemic anticoagulation to be associated with improved outcomes in hospitalized patients the conclusions of such observational studies should be interpreted cautiously in the context of limitations such as incomplete adjustment for confounders and specifically “immortal time bias” [91]. As such data from small observational studies should not be used to guide institutional guidelines in the absence of robust data to suggest a favorable risk benefit profile for such strategies. Figure 1 provides a pragmatic algorithm for the management of anticoagulation in a patient hospitalized with COVID-19 disease based on the limited available evidence.

Besides COVID-19, these patients have multiple other risk factors for development of thrombosis as described above. ICU patients positive for COVID-19 with elevated d-dimer levels and/or clinico-radiological suspicion for thrombosis as noted above should be considered for therapeutic anticoagulation only after careful assessment of their bleeding risk. Choice of agent should be discussed via interdisciplinary consultation and agents selected based on availability, end organ function and administration techniques which emphasize minimization of nursing contact. Active surveillance for thrombosis should continue even after initiation of therapeutic anticoagulation as clot progression has been demonstrated in patients with therapeutic levels of anticoagulation.

Patients with COVID-19 who experience a major thromboembolic event such as PE without any additional risk factors should be considered to have had a “provoked thromboembolic event” and may need 3-6 months of anticoagulation [92]. Minor episodes of DVTs should continue anticoagulation therapy for 2-6 weeks post hospital discharge [93]. The optimal duration of anticoagulation for those with risk factors, either new or preexisting risk factors (eg atrial fibrillation) may need to be modified according to established guidelines [88]. Long term follow up data on thrombotic risk post hospital discharger however remains unclear at this point. Antiviral therapies, which may be utilized in certain COVID-19 patients, are potent enzymes inhibitors and can slow down metabolism and prolong duration of action of many medications including direct oral anticoagulants so caution should be exercised regarding their concomitant dosing [88, 94]. Patients should be comprehensively evaluated by the medical team and pharmacists to determine the most appropriate oral anticoagulant. Prophylactic anticoagulation should be considered in patients presenting with elevated d-dimer levels but with no suspicion or evidence of thrombosis. Decisions on discharge therapy should be based on hospital protocols, patient specific factors, and multidisciplinary discussions regarding the risk benefit profile of chosen strategies.

### Special considerations

The management of anticoagulation in COVID-19 patients extracorporeal membrane oxygenation (ECMO), is even more challenging [95]. Patients with refractory respiratory failure who fail traditional rescue therapies may require veno-venous (VV) ECMO, with a smaller proportion needing veno-arterial (VA) support [95, 96]. Acquired von Willebrand disease, thrombocytopenia, and bleeding are known complications in patients on ECMO [97–100]. Studies have described the use of VV ECMO without anticoagulation to reduce bleeding risks, however, the artificial contact surface of ECMO circuit itself causes continuous activation of coagulation, creating a prothrombotic environment [101, 102]. The thrombotic risk is further accentuated in the presence of uninhibited COVID-19 diseases. Management of COVID-19 patients on ECMO is a balance between need for anticoagulation and risk of bleeding and therefore warrants close multidisciplinary discussions of risk benefit profile. A phase 2 clinical trial is currently underway to assess the efficacy of tPA (tissue plasminogen activator) as salvage therapy for severe ARDS patients (NCT04357730). The consistent demonstration of fibrin in the airspaces and lung parenchyma, along with fibrin-platelet microthrombi in the pulmonary vasculature suggests that plasminogen activators may have a role to limit ARDS progression and reduce ARDS-induced mortality. Currently, the routine use of tPA for salvage in severe ARDS patients is not recommended outside clinical trials until safety and efficacy of this treatment strategy is clearly established.

Pregnant patients with COVID-19 are at higher risk of thrombotic complications. Despite being mostly healthy and young, there are reports of pregnant patients with COVID-19 disease requiring intensive care admissions and being critically ill [103]. With immunocompromised status and physiological adaptive changes during pregnancy, pregnant women could be more susceptible to COVID-19 infection than the general population [104]. The thrombogenic risk of COVID-19 is further exacerbated by pregnancy which by itself is a hypercoagulable condition [105]. Such association of COVID-19 with pregnancy in terms of increasing thromboembolic risk warrants extra caution and requires a multidisciplinary approach to managing anticoagulation in these patients [88].

## Conclusion

Systemic thrombosis is frequently associated with critically ill COVID-19 patients and may lead to fatal outcomes if not diagnosed and managed appropriately. Thrombotic risk commonly persists despite initiation of anticoagulation. Until more information is available, providers should consider prophylactic versus therapeutic anticoagulation based on a combination of patient specific criteria including laboratory results, imaging [88], clinical suspicion and careful balance of thrombotic and bleeding risks. Routine active surveillance guided by clinical and/or radiological assessment is recommended to either pre-empt or aid prompt diagnosis of macrothrombotic events which may be beneficial in guiding anticoagulation strategies. Larger, well designed prospective studies are urgently needed to further elucidate optimal management strategies to mitigate the thrombotic risks associated with COVID-19.

## Data Availability

Not applicable

## Glossary

ARDS: Acute Respiratory Distress Syndrome
COVID-19: Coronavirus disease 2019
PE: Pulmonary embolism
DIC: Disseminated Intravascular Coagulation
ECMO: Extracorporeal Membrane Oxygenation
ROTEM: Rotational Thromboelastometry
EXTEM: Extrinsic pathway of coagulation system abnormality assay using ROTEM
INTEM: Intrinsic pathway of coagulation system abnormality assay using ROTEM
ICU: Intensive Care Unit
IL: Interleukin
MicroCLOTS: Microvascular COVID-19 Lung vessels Obstructive Thromboinflammatory Syndrome
TEG: Thromboelastography
TPA: Tissue plasminogen activator
TNF: Tumor Necrosis Factor
TICP: Thrombocytopenia in Critically ill Patients
SARS-CoV2: Severe Acute Respiratory Syndrome Corona Virus 2
SEC: Spontaneous Echo Contrast
VTE: Venous Thromboembolism
VWF: Von Willebrand factors
